# Putting the pieces together: How is the mitochondrial pathway of apoptosis regulated in cancer and chemotherapy?

**DOI:** 10.1186/2049-3002-2-16

**Published:** 2014-10-06

**Authors:** Rana Elkholi, Thibaud T Renault, Madhavika N Serasinghe, Jerry E Chipuk

**Affiliations:** 1 Department of Oncological Sciences, Icahn School of Medicine at Mount Sinai, One Gustave L. Levy Place, 1425 Madison Avenue, Box 1130, New York, NY 10029, USA; 2 Department of Dermatology, Icahn School of Medicine at Mount Sinai, One Gustave L. Levy Place, Box 1130, New York, NY 10029, USA; 3 The Tisch Cancer Institute, Icahn School of Medicine at Mount Sinai, One Gustave L. Levy Place, Box 1130, New York, NY 10029, USA; 4 The Graduate School of Biomedical Sciences, Icahn School of Medicine at Mount Sinai, One Gustave L. Levy Place, Box 1130, New York, NY 10029, USA; 5 The Diabetes, Obesity, and Metabolism Institute, Icahn School of Medicine at Mount Sinai, One Gustave L. Levy Place, Box 1130, New York, NY 10029, USA

**Keywords:** Apoptosis, BCL-2 family, BH3 mimetics, Cancer, Mitochondria, Oncogenes, Signaling, Tumor suppressors

## Abstract

In order to solve a jigsaw puzzle, one must first have the complete picture to logically connect the pieces. However, in cancer biology, we are still gaining an understanding of all the signaling pathways that promote tumorigenesis and how these pathways can be pharmacologically manipulated by conventional and targeted therapies. Despite not having complete knowledge of the mechanisms that cause cancer, the signaling networks responsible for cancer are becoming clearer, and this information is serving as a solid foundation for the development of rationally designed therapies. One goal of chemotherapy is to induce cancer cell death through the mitochondrial pathway of apoptosis. Within this review, we present the pathways that govern the cellular decision to undergo apoptosis as three distinct, yet connected puzzle pieces: (1) How do oncogene and tumor suppressor pathways regulate apoptosis upstream of mitochondria? (2) How does the B-cell lymphoma 2 (BCL-2) family influence tumorigenesis and chemotherapeutic responses? (3) How is post-mitochondrial outer membrane permeabilization (MOMP) regulation of cell death relevant in cancer? When these pieces are united, it is possible to appreciate how cancer signaling directly impacts upon the fundamental cellular mechanisms of apoptosis and potentially reveals novel pharmacological targets within these pathways that may enhance chemotherapeutic success.

## Review

In multi-cellular organisms, cell growth, cell division, and cell death are regulated by a host of signaling pathways that integrate cellular condition and context. Within healthy tissues, there is a balance between these processes allowing for homeostasis. When this balance is perturbed, usually by uncontrolled proliferation and a collateral failure to activate cell death, susceptibility to cancer is increased. It has been suggested that there are as many ways to cause cancer as there are constellations in the sky—and we highlight a few of these pathways in our discussion below. Despite the many signaling pathways that lead to cancer vulnerability, most would agree that the best method to treat cancer is to specifically eliminate diseased cells via a genetically controlled program of cell death termed apoptosis.

Apoptosis is characterized by cysteine-aspartic protease (caspase)-dependent cleavage of numerous cellular substrates that allows for efficient packaging, detection, and elimination of the targeted cell from the surrounding environment. For our discussion, we will focus on the mitochondrial pathway of apoptosis, which means that mitochondria integrate the pro-apoptotic signaling environment via the B-cell lymphoma 2 (BCL-2) family of proteins to regulate cell death [1,2]. The BCL-2 family controls the integrity of the outer mitochondrial membrane (OMM) and is functionally divided into anti- and pro-apoptotic proteins. Anti-apoptotic BCL-2 members (e.g., BCL-2/BCL-xL/MCL-1) preserve OMM integrity by directly sequestering the pro-apoptotic proteins, which cooperate to form pores with the OMM. Pore formation is referred to as mitochondrial outer membrane permeabilization (MOMP), and this results in the release of mitochondrial proteins (e.g., cytochrome c) that cooperate with cellular adaptor proteins (i.e., APAF-1) to induce caspase activation. From a mechanistic point of view, pro-apoptotic BCL-2 members are further divided into two subclasses: the effectors (e.g., BAX) and BH3-only proteins (e.g., BIM). It is suggested that BAX forms proteolipid pores within the OMM, and this process is nucleated by cooperative interactions with mitochondria and BH3-only proteins [3].

Returning to cancer-causing pathways, there are two general classes of proteins that promote tumorigenesis: oncogenes and tumor suppressors [4]. Oncogenic proteins normally function in homeostatic proliferative and survival mechanisms, but due to mutation (e.g., RAS^G12V^ and BRAF^V600E^) or divergent expression (e.g., *BCL-2*), these proteins undergo a gain of function to promote hyper-proliferation or sustained survival despite pro-apoptotic signaling. Likewise, tumor suppressor proteins (e.g., p53 and PTEN) negatively regulate survival and proliferation following cellular stress, but when mutated or deleted, they fail to appropriately restrain proliferation and this has potential to promote genomic instability and organelle dysfunction. In most cancers, distinct combinations of oncogenic signals and loss of tumor suppressor pathways drive tumorigenesis and resistance to apoptosis. Here, we will discuss specific examples of how oncogenic and tumor suppressor pathways intersect with the apoptotic machinery to alter apoptotic sensitivity, which ultimately impacts upon chemotherapeutic success and patient outcome.

### Piece #1—How do oncogene and tumor suppressor pathways regulate apoptosis upstream of mitochondria?

**Figure 1:**
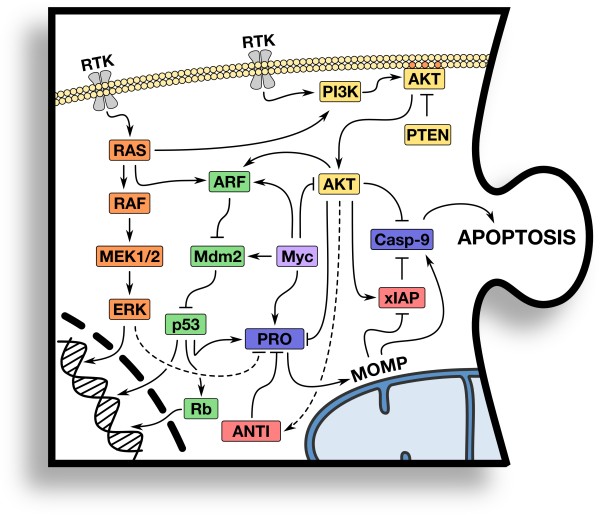
**Piece #1: Tumor suppressor and oncogenic pathways converge on the mitochondrial pathway of apoptosis.** Oncogenic (e.g., PI3K/AKT, RAS-MAPK, and Myc) and tumor suppressor pathways (e.g., p53, PTEN, and Rb) act at transcriptional and non-transcriptional levels to modulate cellular sensitivity to detect and repair stress, along with regulating the expression and function of downstream apoptotic proteins. Details are provided in the text.

Numerous oncogenic and tumor suppressor pathways converge on the BCL-2 family of proteins and downstream effectors to regulate cellular sensitivity to apoptosis during transformation as well as chemotherapeutic interventions. Here, we will discuss some of the most commonly altered tumor suppressor and oncogenic pathways that contribute to apoptotic defects in cancer (Figure 1). The tumor suppressor pathways (p53, retinoblastoma (Rb), and phosphatase and tensin homolog (PTEN)) and the proto-oncogene/oncogene pathways (Phosphoinositide 3-kinase/Akt (PI3K/AKT), RAS/RAF, Myc, and BCL-2) will be highlighted due to their broad implications in multiple tumor types [5,6]. It is important to note that tumor suppressors and oncogenes function via transcriptional and/or non-transcriptional mechanisms. While the p53 tumor suppressor is an example of a protein that functions through both, other proteins such as BCL-2 function primarily through non-transcriptional means at the mitochondria, ER, and perhaps other cellular locations [1]. In the sections that follow, we will discuss these pathways in more detail.

### What is the role of the tumor suppressor network in apoptosis?

One of the major regulators of apoptotic signaling following oncogenic (e.g., aberrant Myc expression) and pharmacological stress (e.g., conventional chemotherapy) is the p53 pathway. p53, often referred to as “guardian of the genome”, is a transcription factor that regulates cellular responses to a multitude of stresses including DNA damage, oncogene activation, and cell cycle and metabolic aberrations [7,8]. In the event of acute stress, the p53 pathway ensures that DNA damage events are allowed to repair prior to mitosis [9-11]. However, if stress is chronic and/or repair mechanisms insufficient, pro-apoptotic signaling mediated by p53 acts to eliminate the affected cell [7].

To commit a cell to apoptosis, p53 acts through both transcriptional and non-transcriptional mechanisms. p53 sensitizes cells to apoptosis through direct transcriptional induction of numerous pro-apoptotic members of the BCL-2 family including, *BAX*, *Noxa*, and *PUMA*[12-15]. In addition to its transcriptional role, p53 directly interacts with multiple members of the BCL-2 family to regulate MOMP. For example, cytosolic and mitochondrial forms of p53 have been shown to directly activate the pro-apoptotic effectors BAK/BAX as well as bind and inhibit the anti-apoptotic proteins BCL-xL and BCL-2 [16-19]. The integration of p53 at multiple points in the mitochondrial pathway of apoptosis highlights the crucial role for this tumor suppressor pathway in the cellular decision to commit to apoptosis.

Another tumor suppressor with highly aberrant expression in many cancers is the Rb protein [20-24]. In unstressed cells, Rb is generally maintained at a hypo-phosphorylated state, which favors a Rb-E2F interaction. During G1, hyper-phosphorylation of Rb by CDK/cyclin complexes disrupts this interaction, thereby de-repressing E2F and allowing for the transcription of genes required for cell cycle progression [25]. Over the years, however, additional anti- as well as pro-survival roles have been described for Rb [26-32]. Consistent with a tumor suppressive function, a pro-apoptotic role for Rb has been described in studies using various cancer cell lines, including glioblastoma, prostate, and cervical cancers [27-29]. In this context, Rb was shown to induce apoptosis in response to genotoxic and oncogenic stresses by promoting transcriptional activation of pro-apoptotic proteins [33]. More recently, Rb was reported to localize to mitochondria and induce apoptosis through direct activation of BAX [34,35]. Interestingly, an anti-apoptotic role has also been described for the protein. Rb has been shown to decrease apoptotic sensitivity in mouse cell lines (again through E2F1 repression) by lowering expression levels of APAF-1 and caspase-9 [31,36,37]. These opposing functions suggest a context-dependent role for Rb in the regulation of apoptosis.

Tumor suppressors are considered the “sentinels” of a cell that protect from oncogenic aberrations to restrict proliferation to healthy cells. Moreover, these pathways function to detect oncogenic stress and/or DNA damage to halt proliferation. It is for these reasons that pre-malignant cells select against this first line of defense in order to initiate tumorigenesis.

### How does oncogenic signaling regulate apoptosis?

A common driver of oncogenesis is the alteration of genes through mutation or chromosomal aberration. While proto-oncogenes ensure a balance between survival and apoptosis to maintain healthy tissues, their mutant form, oncogenes, shifts this balance to favor cell survival, proliferation, and resistance to cell death.

The PI3K/AKT pathway plays a major role in promoting many tumor types. PI3K/AKT is among the most frequently mutated network in cancer [38,39], which leads to massive hyper-activation of this potent survival and proliferation pathway. In addition, several cancers reduce the negative regulator of the pathway, PTEN, a commonly mutated tumor suppressor. PTEN is a dual specificity protein and lipid phosphatase that localizes mainly to the cytosol but is suggested to function in the nucleus and extracellular matrix [40,41]. PTEN negatively regulates the PI3K/AKT pathway by inhibiting the PIP3-dependent activation of AKT [42]. Once active, AKT phosphorylates numerous downstream substrates, including transcription factors as well as direct regulators of apoptosis. Examples of these include the FOXO family of transcription factors which are phosphorylated and inactivated by AKT, resulting in decreased expression of their target pro-apoptotic proteins *BIM* and *PUMA*[43-45]. In addition, AKT directly phosphorylates and suppresses the function of the pro-apoptotic BCL-2 family proteins BAD, BIM, and BAX and upregulates the levels of X-linked inhibitor of apoptosis protein (XIAP) through increased protein stability [43,46-48]. Taken together, the activating mutations in the PI3K/AKT pathway, combined with the inactivation of the PTEN tumor suppressor, result in oncogenic activation of one of the most formidable signaling pathways in cancer. Targeting this pathway at tumor suppressor (i.e., PTEN) and oncogene levels gives the advantage of not only attacking the pro-survival arm of the pathway but also ensuring apoptosis induction through restoration of its tumor suppressor function as well.

The RAS/mitogen-activated protein kinase (MAPK) pathway is another major cellular signaling network that commonly acquires oncogenic mutations at various points in the pathway. For example, mutations in receptor tyrosine kinases (e.g., EGFR, ErbB2), the small GTPase RAS (i.e., RAS^G12V^), and downstream RAF kinases (e.g., BRAF^V600E^) are described in a variety of cancers [38,49-53]. The pathway proceeds via a series of intermediate kinases leading to the activation of extracellular receptor kinase (ERK), which regulates the transcriptional activation of many genes involved in cell cycle and apoptosis. ERK signaling has been shown to transcriptionally activate the pro-survival genes *BCL-2* and *BCL-xL*, as well as stabilize MCL-1 through phosphorylation [54,55]. It has been reported that oncogenic ERK activation leads to a decrease in expression levels of *BIM*, as well as proteasomal degradation of BIM through direct phosphorylation of the protein [56-58]; all of which can be reversed by small molecule inhibition of the pathway [59-62]. In addition, kinases downstream of ERK (e.g., RSK, S6K) directly phosphorylate and inactivate the pro-apoptotic BCL-2 family member BAD, as well as caspase-9 and APAF-1 [63-66].

Myc is a classic oncogenic transcription factor that is over-expressed in a large number of human cancers. Myc expression is upregulated through a variety of mechanisms including chromosomal translocations and amplifications, activation of upstream growth signaling pathways, and increased protein stability [67]. Myc was one of the first proteins identified to have antagonistic pleiotropic functions, promoting both cell survival and cell death [68]. The paradox arises from the oncogene’s ability to cause apoptosis when over expressed, and it has been suggested that this apoptotic phenotype is a measure to ensure protection against unrestricted proliferation, and is bypassed during tumorigenesis [67]. Myc-induced apoptosis can be p53 dependent based on cell type and apoptotic stimulus. Upregulation of p53 by Myc increases the expression of the pro-apoptotic BCL-2 family members, *BAX*, *PUMA*, and *Noxa*[12-15]. Alternatively, a p53-independent mechanism of Myc exists by either directly suppressing *BCL-2* expression in a cell type-specific manner or directly acting on *BIM* expression [69]. More recently, the oncometabolite 2-hydroxyglutarate from isocitrate dehydrogenase mutant cancers was found to directly activate Myc-mediated apoptosis in breast cancer [70], suggesting that Myc may be an important link between altered cellular metabolism and apoptosis in cancer.

The focus of this section thus far has been on how potent oncogenes function to ensure cell survival and target apoptotic pathways to reduce cell death sensitivity. Last but not least on this list comes the founding member of the BCL-2 family itself. Originally identified as a chromosomal translocation in B-cell lymphoma, BCL-2 is the founding member of the family that is responsible for directly inhibiting the mitochondrial pathway of apoptosis [71]. The translocation identified in B-cell lymphoma positions *BCL-2* under the control of the immunoglobulin heavy-chain promoter, leading to massive *BCL-2* over-expression and subsequent resistance to cell death. The function of BCL-2 as an oncogene is unusual in that over-expression alone is not sufficient to drive cellular transformation but requires additional oncogenes (e.g., Myc) [72]. This result revealed that BCL-2 does not promote cell proliferation, but rather it blocks pro-apoptotic signals from collateral oncogenes. While the example of *BCL-2* translocation in lymphoma is not observed in many tumor types, over-expression of anti-apoptotic members of the BCL-2 family is a common feature in cancers of the uterus, lung, ovary, breast, colon, liver, and gastrointestinal tract [73-76]. The mechanism by which BCL-2 expression directly controls apoptosis will be discussed shortly.

The oncogenic and tumor suppressor pathways mutated in cancer have become major targets for drug development over the past few decades. While most conventional chemotherapy responses proceed via the mitochondrial pathway of apoptosis (often mediated by DNA damage and p53), more recently there has been explicit focus on the development of targeted therapies for specific proteins within these tumorigenic pathways. Table 1 presents a sampling of the current and developing drugs targeting the tumor suppressors and oncogenes described above. While tumor suppressor and oncogenic pathways require mitochondrial contributions to die, the cellular decision to initiate MOMP and apoptosis is governed by the functional repertoire of BCL-2 family proteins at the OMM. In the next section, we will discuss how the BCL-2 family of proteins impacts upon the execution of the mitochondrial pathway of apoptosis in response cancer cell signaling and chemotherapeutics.

**Table 1:** Drugs currently in clinical trials targeting tumor suppressor/oncogene pathways or proteins within the mitochondrial pathway of apoptosis

	**Target**	**Drug**	**Mechanism**	**Clinical trial**
Tumor suppressors/oncogenes	p53	ADVEXIN (Ade5CMV-p53)^1^	Gene therapy for introduction of wtp53	Phase III
	P13K	Idelalisib (GS-1101)^2^	Inhibitor of PI3Kδ	Phase II
		Buparlisib^3^	ATP competitive inhibitor of class I PI3K	Phase II
		SAR245408 (XL 147)^4^	ATP competitive inhibitor of class I PI3K	Phase I/II
	P13K/mTOR	BEZ235^3^	Dual kinase inhibitor to PI3K and mTOR	Phase II
		BGT226^3^	Dual kinase inhibitor to PI3K and mTOR	Phase I
		PF-04691502^5^	Dual kinase inhibitor to PI3K and mTOR	Phase I/II
		SAR245409^6^	Dual kinase inhibitor to PI3K and mTOR	Phase II
	AKT	Perifosine^7^	Inhibitor to AKT	Phase I/II
	Receptor tyrosine kinases (e.g., EGFR)	Iressa (Gefitinib)^8^	ATP competitive tyrosine kinase inhibitor	Phase I/II
		Tarceva (Erlotinib)^9^	ATP competitive EGFR inhibitor	Phase II/III
		Cetuximab^10^	Monoclonal-antibody against EGFR prevents receptor dimerization	Phase II/III
		Tykerb (Lapatinib)^11^	Inhibitor to receptor phosphorylation	Phase I/II
		Vectibix (Panitumumab)^12^	Monoclonal antibody against EGFR inhibits receptor activation	Phase II
	RAS	Salirasib^13^	Blocks RAS membrane association	Phase II
		Sarasar (Lonafarnib)^14^	Inhibitor to farnesyl transferase	Phase II
		Zarnestra (Tipifarnib)^15^	Inhibitor to farnesyl transferase	Phase II/III
	BRAF^V600E^	Zelboraf (Vemurafenib)^9^	ATP-competitive selective inhibitor	Phase II
	RAF	Nexavar (Sorafenib)^16^	Multi-kinase inhibitor	Phase II/III
		Tafinlar (Dabrafenib)^11^	ATP competitive kinase inhibitor	Phase I/II
	MEK	Mekinist (Trametinib)^11^	MEK inhibitor	Phase II/III
Mitochondrial pathway	Anti-apoptotic BCL-2 proteins	Navitoclax (ABT-263)^17^	Inhibits BCL-2, BCL-w, and BCL-xL	Phase I/II
		ABT-199^17^	Inhibits BCL-2	Phase I
		Gossypol(AT-101)^18^	Inhibits BCL-2, BCL-xL, MCL-1 and BCL-w	Phase I/II
		Obatoclax^19^	Inhibits BCL-2, BCL-xL, and MCL-1	Phase I/II
	XIAP	GEM640 (AEG35156)^20^	Blocks expression of XIAP	Phase I/II
	IAPs	LCL-161^3^	Peptidomimetic of SMAC-inhibits IAPs	Phase I/II
		Birinapant (TL32711)^21^	Peptidomimetic of SMAC-inhibits IAPs	Phase II

Records were obtained from the National Cancer Institute and NIH clinical trials databases (http://www.cancer.gov; https://clinicaltrials.gov).

^1^Introgen Therapeutics, TX, USA, ^2^Gilead, CA, USA, ^3^Novartis, Basel, Switzerland, ^4^Exelixis, CA, USA, ^5^Pfizer, NY, USA, ^6^Sanofi, Paris, France, ^7^Aeterna Zentaris, Quebec, Canada, ^8^Astra Zeneca, London, UK, ^9^Genentech, CA, USA, ^10^Imclone Systems Inc., NY, USA, ^11^GlaxoSmithKline, Middlesex, UK, ^12^Amgen, CA, USA, ^13^Concordia Pharmaceuticals, FL, USA, ^14^Merck, NJ, USA, ^15^Johnson & Johnson, NJ, USA, ^16^Onyx Pharmaceuticals, CA, USA, ^17^AbbVie, IL, USA, ^18^Ascenta Therapeutics, PA, USA, ^19^Gemin X Pharmaceuticals, Quebec, Canada, ^20^Aegera Therapeutics, Quebec, Canada, ^21^TetraLogic Pharmaceuticals, PA, USA.

### Piece #2—How does the BCL-2 family influence tumorigenesis and chemotherapeutic responses?

**Figure 2:**
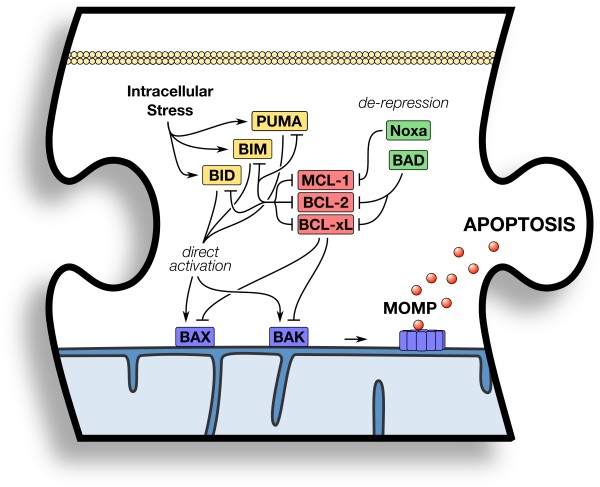
**Piece #2: The BCL-2 family controls BAK/BAX activation and MOMP.** Pro-apoptotic BCL-2 family protein activation is triggered by extra- and intra-cellular signaling. De-repressor BH3-only proteins (*green*) prevent or disrupt inhibition by anti-apoptotic proteins (*red*). Direct activator BH3-only proteins (*yellow*) bind BAK and BAX (*blue*) to induce their homo-oligomerization and MOMP. Details are provided in the text.

The BCL-2 family comprises four groups of proteins, which are based on their domain composition (i.e., one to four BCL-2 homology “BH” domains) and function [77]. Pro-apoptotic effectors (BAK and BAX) are mechanistically involved in MOMP as these proteins interact with the OMM, leading to its permeabilization [77]. In order for BAK or BAX to initiate MOMP, they must be localized to the OMM, which creates the appropriate biophysical and biochemical environment to support structural rearrangements that nucleate homo-oligomerization into proteolipid pores [78,79]. The structural rearrangements can be triggered by various mechanisms, but the one most relevant to our discussion is via protein-protein interactions with the direct activator BH3-only proteins (e.g., BID and BIM) [80-82]. Through direct binding of these proteins to either the N-terminus of BAK/BAX or a hydrophobic region in the core of the protein formed by the BH1–3 domains, the direct activators trigger essential conformational changes that render these effector proteins competent to oligomerize [82-84]. Conversely, the anti-apoptotic proteins (e.g., BCL-2, BCL-xL/BCL2L1, BCL-w/BCL2L2, A1/BCL2A1, and MCL-1) inhibit BAK/BAX activation by direct interaction or by sequestration of the direct activator BH3-only proteins [80,81]. This inhibition can be prevented (termed “sensitization”) or displaced (termed “de-repression”) by the final group of BCL-2 family members, the sensitizer/de-repressor BH3-only proteins (e.g., BAD, Noxa, and PUMA) [80,85]. As the role of the BH3-only proteins is significant in the regulation of cellular sensitivity to apoptosis via BAK and BAX activation, we will discuss several approaches to regulate BH3-only protein function by intracellular signaling pathways and small molecules that were designed to mimic their action. The mechanism of action of the BCL-2 family to regulate apoptosis is summarized in Figure 2.

### BCL-2 family deregulation in cancer

The regulation of MOMP is complex due to multiple proteins and pathways converging upon the BCL-2 family; furthermore, there are specific expression and functional patterns that are dependent upon cell type and differentiation state [86]. What is key to understanding how the BCL-2 family regulates apoptosis in cancer is directly linked to the mechanisms described earlier, those being sensitization, de-repression, and direct activation of BAK/BAX. In addition to the above *BCL-2* translocation event, epigenetic regulation of anti-apoptotic BCL-2 proteins also plays a role in reducing cellular sensitivity to apoptosis. As an example, hypo-methylation of the *BCL-2* promoter has been reported in chronic lymphocytic leukemia (CLL) [87]. Of course, the expression of anti-apoptotic proteins is positively selected during transformation because the targeted cell is trying to eliminate itself through pro-apoptotic signaling, yet oncogenic and tumor suppressor pathways must promote anti-apoptotic BCL-2 family function to survive [88]. The dual upregulation of pro-apoptotic and anti-apoptotic proteins is referred to as “priming”, which means the cells are uniquely poised to engage apoptosis due to constitutive sequestration of pro-apoptotic proteins, such as BIM. The presence of sequestered BIM presents a pharmacological opportunity to treat primed cancer cells with BH3 mimetics (discussed below) as pro-apoptotic signaling appears intact [89,90].

Post-transcriptionally, several cancer-associated miRNAs are involved in the control of the BCL-2 family. For example, miR-15a and miR-16-1 are reduced in about two thirds of B-cell CLL cases resulting in *BCL-2* over-expression and the establishment of disease [91]. Other miRNAs in CLL, such as miR-181a/b, attenuate *BCL-2* and *BCL-xL* expression and are markers of chemotherapeutic success [92]. In addition to regulation at the transcriptional and translational levels, members of the BCL-2 family are controlled by a variety of post-translational modifications. For instance, BAD phosphorylation on serines 112 and 136 is exacerbated in glioblastomas, prostate cancers, and melanomas due to a combination of oncogenic MAPK signaling and PTEN mutation/downregulation [93]. This situation likely mediates sensitivity to apoptosis by altering the affinity of BAD for anti-apoptotic partners, thereby influencing sensitization and de-repression mechanisms. On a similar note, BIM-EL (one of three BIM isoforms) phosphorylation at serine 69 by oncogenic MAPK signaling influences associations with MCL-1 and correlates with resistance to apoptosis in CLL [94]. Altogether, the above examples show that the BCL-2 family proteins are regulated at the genomic, translational, and post-translational levels by cancer-associated pathways.

### How do we pharmacologically target the BCL-2 family?

In order to engage apoptosis, BH3-only proteins must interact with anti- and pro-apoptotic BCL-2 proteins; therefore, the majority of small molecules identified to regulate apoptotic sensitivity mimic these interactions. One of the first natural BH3-mimetic molecules discovered was gossypol, a polyphenol extracted from cottonseed [95]. Gossypol and its derivative, TW-37, and apogossypolone (ApoG2) target BCL-2, BCL-xL, and MCL-1, and effectively promote apoptosis in lung, prostate, and lymphoma cancer models [96-98]. In parallel to naturally derived compounds, numerous small molecules were engineered through structure activity relationship strategies to target the hydrophobic groove of anti-apoptotic BCL-2 proteins. For example, chemical engineering and assembly of several low affinity molecules led to the generation of the highly specific drug ABT-737 [99]. Despite lacking some key pharmacological properties required to be used in the clinic (*e.g.,* non orally bio-available), the discovery of ABT-737 constituted a milestone in specifically targeting the BCL-2 family, and further modification of this drug led to the bioavailable derivative, ABT-263. ABT-737 and ABT-263 are highly specific for BCL-2, BCL-xL, and BCL-w and have proven efficacy on BCL-2/BCL-xL-dependent tumors such as leukemia and lymphoma [99]. As an aside, one commonly observed side effect of ABT-263 therapy is rapid thrombocytopenia, which occurs because platelets rely exclusively on BCL-xL for survival [100,101]. To avoid this phenotype, an additional derivative (ABT-199) was generated that has markedly reduced its affinity for BCL-xL, and is therefore more specific to BCL-2 [102]. Indeed, ABT-199 was shown to retain the same efficiency as ABT-737 on leukemia and lymphomas without the collateral thrombocytopenia [102,103].

Consistent with the development of rational drug design to target anti-apoptotic BCL-2 proteins, new molecules were recently reported, including MIM1 [104], Terphenyl-14, and WEHI-539 [105,106], which specifically target MCL-1 and BCL-xL, respectively. These pharmacological agents are highly significant to designing precision treatments as tumors frequently display dependency upon BCL-2 or MCL-1, and chemo-resistant tumors often shift reliance between anti-apoptotic BCL-2 proteins. Importantly, the dependency upon different anti-apoptotic BCL-2 proteins can be determined by BH3 profiling to reveal which patients are most likely to respond to conventional chemotherapy [107,108]. Interestingly, recent evidence suggests that response to BH-3 mimetics is not only determined by the anti-apoptotic proteins, but the pro-apoptotic repertoire as well [109]. In addition to targeting anti-apoptotic BCL-2 members, recent BH3-mimetics design has generated small molecules that function similar to direct activator BH3-only proteins to directly induce BAX activation and MOMP. For example, the small molecule BAX activator molecule 7 (BAM7) demonstrates impressive potency to activate BAX, similar to BIM in transformed cells [110]. Of course, one relevant question is how will this novel class of molecules be used to specifically kill cancer cells? It is likely that novel combinations of sub-threshold levels of chemotherapeutics will provide the best patient benefits.

As discussed earlier, BH3 mimetics are useful as single agents in hematological malignancies harboring BIM; however, the majority of solid tumors do not constitutively express direct activator BH3-only proteins [111]. Therefore, the design of combination therapies must incorporate strategies to induce direct activator BH3-only protein expression in order to sensitize solid tumors to BH3 mimetics. For example, inhibition of BRAF^V600E^ signaling by PLX-4032 triggers a stress response that leads to increased expression and accumulation of BIM at the OMM [61,112]. These sequestered molecules of BIM can be functionalized by the collateral inhibition of the anti-apoptotic BCL-2 repertoire using ABT-737 for example [61]. Similar approaches using conventional chemotherapies have generated comparable results, suggesting broad applications for these therapeutic strategies [113].

### Piece #3—How is post-MOMP regulation of cell death relevant in cancer?

So far, we discussed the various cancer-related signaling pathways upstream of mitochondria taking into consideration the dynamic interactions within the BCL-2 family at the OMM that lead to the decision to die. Following MOMP, a cell normally enters the final stages of demise; while this is often considered the “point of no return”, there is a growing literature suggesting cells maintain the ability to resist cell death despite caspase activation [114]. Here, we will highlight several cellular mechanisms that regulate cell fate post-MOMP including intermembrane space (IMS) protein release, caspase activation, and cellular clearance. The therapeutic opportunities to target these final stages of apoptosis will also be discussed.

### What happens post-MOMP?

**Figure 3:**
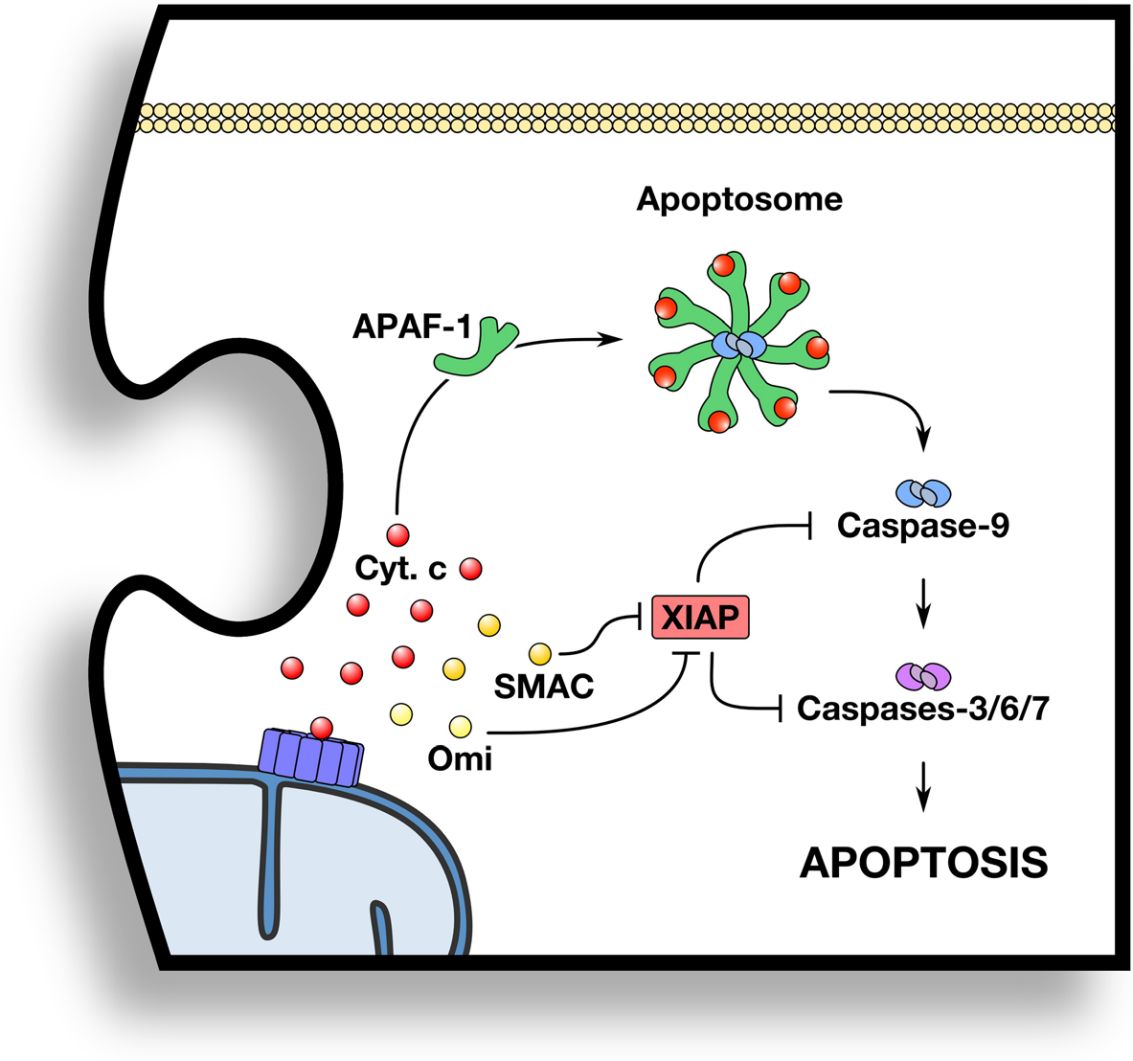
**Piece #3: Post-MOMP regulation of cell death.** Pro-apoptotic proteins within the mitochondrial inter-membrane space (e.g., cytochrome c, SMAC, and Omi) are released after MOMP and directly regulate the activation of caspases and commitment to apoptosis. Details are provided in the text.

Once BAK/BAX homo-oligomers permeabilize the OMM, the inner mitochondrial membrane (IMM) and cristae junctions undergo extensive remodeling [115-117]. This remodeling allows several IMS proteins to diffuse into the cytosol (e.g., cytochrome c, SMAC, Omi, and nearly all soluble IMS proteins). One of the most crucial of these proteins is cytochrome c, which binds the cytosolic adaptor protein apoptotic protease activating factor (APAF-1) and triggers the formation of a heptameric complex that recruits and activates procaspase-9. Activated dimeric caspase-9 directly cleaves and activates the downstream effector caspase-3 and caspase-7 (Figure 3). These effector caspases are responsible for eliciting the classical apoptotic phenotypes characterized by DNA laddering, phosphatidylserine exposure, and contraction from the surrounding healthy cells. The cellular phenotypes stemming from caspase activation also allow for the generation of “find me” and “eat me” signals that elicit phagocytosis and removal of the dying cell [118,119].

As we deepen our mechanistic understanding of how the mitochondrial pathway of apoptosis proceeds after MOMP, the majority of the literature would agree with the notion that irrespective of caspase activation (i.e., caspase activation promotes rapid packaging and detection, but the inhibition of caspases will only delay, not prevent cell death), most cells die following MOMP due to aberrations in mitochondrial biology. However, the general applicability of this concept is increasingly being called into question. Genetic evidence unquestionably supports a pro-survival role for cytochrome c as an integral part of the electron transport chain; cytochrome c knockout mice are embryonic lethal due to an organism-wide failure to generate ATP, and tissue-specific deletions of cytochrome c corroborate these results [120,121]. Somewhat paradoxically, cytochrome c also functions as a crucial mediator of caspase-dependent death; cells deficient in cytochrome c are resistant to cytotoxic insults [122]. Whole organism or tissue-specific deletion eliminates cellular and/or tissue viability, presumably through a reduction in ATP generation and developmental cell death that is required for tissue and organ function. Interestingly, developmental phenotypes are shared with downstream apoptotic counterparts. *Apaf1* and *Caspase9* deficiencies result in the inhibition of developmental apoptosis, with phenotypes most usually characterized by exencephaly and cranioschisis [123,124]. The function of these proteins in tumor suppression however remains controversial. While genetic studies have shown that *Apaf-1* and/or *caspase-9* deletion promote Myc-induced oncogenic transformation of MEFs, *in vivo* deletion of these genes reportedly had no effect on the rate, severity, or chemotherapeutic response of Myc-induced lymphomas [125,126]. This is in contrast to what is observed, for example, with deletions of pro-apoptotic proteins such as *BIM* and *BAD*. Deletions of either of these genes have been shown to enhance Eμ-*myc* induced lymphoma, highlighting their importance in suppressing lymphomagenesis [127,128].

To ensure that pro-apoptotic caspases are not inappropriately activated in unstressed cellular conditions, additional “apoptotic brakes” are in place that prevent caspase activation [129]. One example is XIAP, which promotes cellular survival by inhibiting caspase activation via direct protein-protein interactions [130]. Following MOMP, the anti-apoptotic activity of XIAP is counteracted by the release of two IMS proteins: second mitochondria-derived activator of caspase (SMAC) and Omi/Htra2 (Omi). Once released into the cytosol, SMAC and Omi bind and antagonize the activity of XIAP, thereby allowing for caspase activation to proceed [131,132]. The function of SMAC and Omi suggests that post-MOMP regulation of caspase activity is required, which would not be the case if MOMP was always sufficient to promote death. It is important to mention that the *Smac* and *Omi* knockout mice develop normally and exhibit no defects in susceptibility to apoptosis [133]. This suggests a possible redundancy in the function of these proteins or a specificity in cellular stress conditions. Despite an unclear role in apoptosis, both of these proteins also have been suggested to play a role in cancer progression and chemotherapeutic responses. A decrease in SMAC expression, at the mRNA and protein levels, has been reported in many malignancies including renal cell carcinoma, hepatocellular carcinoma, testicular cancer, and lung cancer. Interestingly, in many of these studies, a decrease in SMAC levels was also accompanied by an increase in inhibitor of apoptosis protein (IAP) expression level as well as an increase in tumor invasion and metastasis [134-138].

It has become evident that in many cell types, there is an anti-apoptotic threshold for endogenous caspase activation, as well as XIAP levels, to modulate cell death responses. This notion is supported by the observation that irradiation of cells leading to permeabilization of up to 15% of the mitochondrial population does not induce an apoptotic response, suggesting that local release of mitochondrial proteins does not result in an amplifiable apoptotic signal [139]. This may also explain the contribution of XIAP over-expression in many tumors, thereby increasing the threshold for caspase activation and efficient execution of cell death. It is worth mentioning that *XIAP* knockout mice are viable and lack apoptotic defects. These mice do, however, show increases in cellular IAP (c-IAPs) protein levels suggesting that these proteins may compensate for XIAP loss during development and apoptosis [140].

The ultimate goal of post mitochondrial regulation of pro-apoptotic BCL-2 family function and MOMP is to initiate the activation of caspases that will complete the apoptotic program. It is important to consider however that while caspases play a role in mediating cell death, they also play important roles in maintaining cell survival. Caspases generally thought to function exclusively in apoptosis are now being reported to have many additional cellular functions [141]. Executioner caspases have been shown to play roles in adaptive immunity as well as cell fate decisions including cell differentiation and migration [142-145]. This raises the question of how a cell can differentiate between apoptotic and non-apoptotic caspase activation. Studies have suggested that a threshold of caspase activation exists in cells where only small levels of activation are required for non-apoptotic functions, whereas much higher levels are required to execute cell death. Another possible mechanism of regulated non-apoptotic caspase activation is the compartmentalization of active caspases. Examples of such mechanisms have been demonstrated in neurons, as well as in macrophages where caspase containing inflammasomes have been shown to form. In cancer, overall levels of caspases, particularly executioner caspases, can be expressed at very low levels. A screen of primary breast tumors found that approximately 75% of tumors lacked *CASP3* transcript as well as protein expression [146], and similar findings were reported in colorectal and gastric tumors, which were found to express very low or absent levels of caspase-7 [147,148]. It is important to mention however that due to the redundancy of these proteins, very little evidence supports a role for individual caspases in regulating tumorigenesis. Individual caspase knockout animals exhibit quite mild phenotypes and cells derived from these mice are only slightly more resistant to apoptosis than their WT counterparts. Cells lacking both *CASP3* and *CASP7* however are extremely resistant to apoptotic stimulus [149]. These observations raise the possibility that low levels of caspase activation may promote cell survival and/or tumorigenesis. Among the demonstrated non-apoptotic roles of caspases is role in cell migration and potentially invasiveness [150,151]. It is possible that low or basal levels of caspases promote cellular migration to a more tumor favorable milieu.

### Is there regulation of apoptosis after MOMP?

Given the indication that several mechanisms are in place to regulate caspase activation and apoptosis post-MOMP, the next question that arises is why a cell would need to commit resources to do so once mitochondrial integrity has been compromised. As previously mentioned, it appears that a specific threshold of cytochrome c release and subsequent caspase activation must be reached in order to elicit an apoptotic response. This may be a mechanism to ensure that a cell survives any potential “accidental MOMP” events. Recovery post-MOMP may also be essential for post-mitotic cells including cardiomyocytes and sympathetic neurons. Such tissues exhibit poor regenerative potential and therefore have adapted mechanisms to ensure longevity despite incomplete MOMP [152,153]. Lower APAF-1 levels have been reported in both cell types as well as resistance to cytochrome c microinjection. Inhibition of XIAP through the addition of recombinant SMAC or deletion of *XIAP* resensitizes these cells, which further highlights the importance of XIAP in maintaining cellular survival [154-156].

Finally, ensuring regulation of cell death post-MOMP is essential for recovery in proliferating cells and has important implications for tumorigenesis. As discussed throughout this section, tumors have been shown to develop mechanisms such as loss of APAF-1, defective caspase activation, and upregulation of XIAP to bypass complete cell death [157-161]. Cancer-associated pathways like PI3K/AKT have been shown to antagonize caspase activity by phosphorylation of caspase-9 and caspase-3 [151,162]. The cellular mechanisms related to caspase inhibition post-MOMP may present interesting therapeutic opportunities that can be exploited for cancer treatment.

### Can cells survive despite MOMP?

As discussed, cytochrome c is not only essential for apoptosome formation but is also an essential component of the electron transport chain. Once MOMP has occurred and cytochrome c is released, not only does this trigger the apoptotic cascade but also transiently shuts down the electron transport chain. One would expect that both these events would effectively render cell survival post MOMP unlikely; however, there is evidence of scenarios where cells do recover and survive. This paradox raises the question of how cells can survive once MOMP has occurred. Interestingly, a study by Colell et al. implicated Glyceraldehyde 3-phosphate dehydrogenase (GAPDH) in mediating cellular recovery following MOMP. The authors showed that through enhanced glycolysis and autophagy, GAPDH could mediate clonogenic survival post-MOMP if caspase activation was inhibited [163]. In addition, work by Tait et al., in 2010 demonstrated that often, cells undergo incomplete MOMP. Through live cell imaging, it was determined that not all mitochondria in a cell undergo MOMP in response to apoptotic stimulus. The small surviving population provides a cohort of intact, healthy mitochondria that can potentially repopulate the mitochondrial network and allow for full cell recovery [164]. Not only do these studies demonstrate how cells could potentially survive once MOMP has occurred but also they further underscore the importance of caspases in mediating apoptosis. While these studies propose interesting mechanisms of post-MOMP recovery of cells, the significance of these processes has yet to be explored in a tumorigenic setting.

### How can post-MOMP events be targeted for therapeutic purposes?

The majority of cell death research with direct implications on killing cancer cells has focused on the identification of pathways and therapeutics that promote apoptosis at the levels of pro-apoptotic signaling (Piece #1) and the BCL-2 family (Piece #2). Given that many tumors have adapted mechanisms to reduce apoptosis by regulating activities following MOMP, targeting post-mitochondrial proteins may present novel therapeutic opportunities.

In 2000, the first crystal structure of the interaction between SMAC and IAPs was reported [165-167]. This structure served as the basis for the development of SMAC mimetics to act as IAP antagonists. These peptides have been shown to effectively inhibit IAP activity in several cancer cells, thereby sensitizing them to pro-apoptotic stimuli [168]. In non-small cell lung cancer, SMAC mimetic JP1201 was shown to sensitize cells to standard chemotherapy [169]. The same peptide was also shown to reduce primary and metastatic tumor burden in xenograft models of pancreatic cancer when used in combination with chemotherapeutics [170]. Interestingly, not only do these molecules sensitize cells to mitochondrial apoptosis through XIAP degradation but also to TNF-induced cell death by antagonizing cellular IAPs. Indeed, SMAC mimetics can sensitize to inducers of non-apoptotic cell death via the regulation of TNF receptor mediated signaling, and this is also influenced by pro-survival pathways, such as NFκB [171]. Several other SMAC mimetics have also been developed and are beginning to show efficacy in phase I and II clinical trials (see Table 1). In addition to SMAC mimetics, several IAP antagonists have been developed, including specific XIAP and cIAP inhibitors as well as XIAP antisense oligonucleotides. The latter has shown promising effects in phase I and II clinical trials when used in combination with standard chemotherapy in patients with acute myeloid leukemia [172].

## Conclusions

The focus of our discussion has been to describe the numerous mechanisms by which tumor suppressor and oncogenic pathways reduce apoptotic sensitivity to initiate tumorigenesis and how these aberrations ultimately impact upon the success of chemotherapeutic interventions. From the evidence provided above, it appears that there are two pro-apoptotic signaling networks that may be specifically disrupted to ensure the survival of cells harboring oncogenic signals (e.g., oncogenic MAPK signaling) or genomic instability (e.g., DNA lesions). The first being upstream of the core apoptotic machinery; this includes the proteins and pathways (e.g., the p53 pathway) that specifically detect and respond to oncogenic signaling and macromolecular damage. When these pathways fail to recognize aberrations, the compromised cell does not initiate cell cycle arrest and repair mechanisms to maintain stability. In situations of chronic or irreparable cellular stress, a cell may be able to detect cellular damage, but if the pro-apoptotic machinery is not effectively engaged to eliminate the compromised cell (e.g., *BCL-2* over-expression), its persistence increases the likelihood of developing and maintaining secondary events that may initiate malignancy, and potentially, chemotherapeutically intractable disease.

Since the advent of cancer chemotherapy, conventional treatments that promote apoptosis (e.g., cisplatin, dacarbazine, vinblastine) have provided the bulk of positive patient responses and remissions, yet the negative side effects and low response rates for many tumor types force scientists and clinicians to search for more optimal strategies. Given our broader knowledge of how the above pathways function in both physiological and pathophysiological apoptosis, it is being increasingly evident that pharmacologically targeting the specific upstream (e.g., BRAF^V600E^) and/or direct pro-apoptotic signaling pathways (e.g., BH3 mimetics) will likely provide a patient benefit. Returning to the jigsaw puzzle analogy mentioned earlier, our discussion on the three key distinct steps (or puzzle pieces) that regulate apoptotic sensitivity before and after chemotherapeutic interventions reveals that we are making significant progress in understanding the key contributions of apoptosis in cancer and chemotherapy. Likewise, as we continue to identify mutations and mechanisms that directly control apoptosis and malignancy, our pharmacological space to rationally design small molecules will hopefully allow for enhanced precision medicine to specifically eradicate malignant cells.

## Competing interests

The authors declare that they have no competing interests.

## Authors' contributions

RE, TTR, MNS, and JEC discussed and wrote the manuscript. All authors read and approved the final manuscript.
